# Mobile Apps for Heart Rate Variability: App Store Search and Content Analysis

**DOI:** 10.2196/84764

**Published:** 2026-07-17

**Authors:** Eline de Jager, Brian Caulfield, Evgenia Angelidi, Sinead Holden

**Affiliations:** 1 School of Public Health, Physiotherapy, and Sports Science, University College Dublin, Belfield, Dublin, Ireland, +35317163432; 2 Insight Research Ireland Centre for Data Analytics, Dublin, Ireland; 3 UCD Institute for Sport and Health, University College Dublin, Dublin, Ireland

**Keywords:** smartphone apps, photoplethysmography, digital health, wearable electronic devices, mobile health, mHealth, autonomic nervous system, devices

## Abstract

**Background:**

Heart rate variability (HRV) is a noninvasive indicator of autonomic nervous system activity that is increasingly used for health and performance monitoring. Digital and mobile technologies are increasingly providing opportunities for remote HRV monitoring outside of laboratory-based settings.

**Objective:**

This study aimed to describe the landscape of mobile apps that measure, analyze, and provide feedback on HRV, with a focus on how HRV is measured, analyzed, interpreted, and communicated to users. A secondary aim was to assess the transparency of these apps, including the extent to which they disclose the evidence underpinning their HRV metrics and feedback.

**Methods:**

This study was an app store search and content analysis. Searches were conducted in the Google Play Store and Apple iTunes Store. Apps were eligible for inclusion if they had functionality to record, analyze, or provide feedback on HRV and were available in English. Data were extracted from app descriptions, screenshots, websites, and, where necessary, contact with developers. Data were extracted on app metadata (developer, release and update dates, and pricing), alongside information about HRV measurement, analysis, and feedback. This included the type of sensor used; HRV measurement characteristics (sensor placement, recording duration, and body position or standardization procedures); methods to calculate and interpret HRV (ie, metrics derived and how they were interpreted for users); and additional app functionality such as reminders, the ability to log self-reported stressors, and the type of feedback or guidance provided based on HRV. We used previously published criteria for assessing the quality of information on the internet, which included authorship, scientific attribution, currency of updates, and data privacy.

**Results:**

Of 746 apps identified, 206 met eligibility criteria. Of these, 132 were primary measurement apps, 59 were aggregators, and 15 were hybrid. Photoplethysmogram was the most common sensing modality (n=117, 56.8%), followed by multiple sensors (n=60, 29.1%). Full data extraction across app metadata and HRV measurement and analysis data was only achievable for 93 (45.1%) apps, representing a transparent subset with sufficient available information for content analysis. The most commonly reported HRV metrics were root mean square of successive differences (n=51) and SD of normal-to-normal intervals (n=48), while frequency-domain power (n=22) and low frequency to high frequency ratios (n=15) were less common. Most apps presented data as personalized trends or individualized ranges (76/93, 81.7%), emphasizing user-specific context rather than isolated values. Although 86% (80/93) offered contextual guidance (eg, readiness or recovery scores), many relied on proprietary algorithms that were not transparently described, limiting independent assessment of how these scores were derived and validated.

**Conclusions:**

Consumer HRV apps are widely available but vary considerably in how data are collected, processed, and contextualized. While many offer personalized trends and guidance, methodological transparency is often limited, particularly regarding the proprietary algorithms underlying the feedback scores.

## Introduction

Heart rate variability (HRV) is the variation in time intervals between consecutive heartbeats [1,2]. This metric is a noninvasive indicator of autonomic nervous system activity, reflecting the balance between the sympathetic (fight-or-flight) and parasympathetic (rest-and-digest) branches [1,2]. Generally, higher HRV is associated with greater parasympathetic influence, indicating effective recovery, adaptability, and overall cardiovascular health [3-5]. Conversely, lower HRV is often linked to increased sympathetic activity, which may result from physical stress, intense training, illness, or other physiological and psychological factors [3-5]. HRV metrics have been shown to correlate with a range of health outcomes, including cardiovascular fitness, stress levels, and overall well-being [3-5].

The gold standard for measurement of HRV is an electrocardiogram (ECG), with traditional assessment protocols ranging from 5 minutes to 24 hours [1]. Many smartphone apps now use photoplethysmography (PPG) technology to estimate HRV by detecting changes in blood volume associated with each heartbeat through light reflected from the skin and captured by smartphone cameras or wearable sensors [6]. This enables an estimate of HRV through the pulse rate waveform. This has made HRV monitoring highly accessible to the general public. Advances in signal processing have enabled PPG to achieve reasonable accuracy under resting conditions [7-9]. However, this accessibility has created a “transparency gap”: while many apps provide sophisticated feedback on stress and readiness, the underlying measurement protocols and algorithms often remain proprietary and undisclosed.

A major concern in the current ecosystem is not necessarily the technical accuracy of the sensors themselves but the transparency of the evidence base used to justify app-provided insights. Many apps cite peer-reviewed literature to establish credibility; however, it is often unclear whether these citations support the general physiological theory of HRV or if they represent specific disclosure of the app’s own methodology. Without clear reporting of how metrics are calculated and what evidence supports them, users cannot distinguish between scientifically grounded tools and those using unverified metrics [10].

To address this gap, this study does not seek to validate app measurements but rather to audit the transparency and scientific attribution of the HRV app market. This study aims to describe the landscape of mobile apps that measure, analyze, and provide feedback on HRV, with a focus on how HRV is measured, analyzed, interpreted, and communicated to users. A secondary aim is to assess the transparency of these apps, including the extent to which they disclose the evidence underpinning their HRV metrics and feedback.

## Methods

### Study Design and Search

This study is designed as an app store review and content analysis of smartphone apps designed to measure or monitor HRV. The reporting of this study follows the Quality and Risk of Bias Checklist for Studies That Review Smartphone Applications [11] and the PRISMA (Preferred Reporting Items for Systematic Reviews and Meta-Analyses) statement guidelines [12]. A search of HRV apps was conducted in the Google Play Store (Android) and iTunes App Store (iOS) between October and November 2024 [13]. These account for approximately 99.3% of smartphone operating systems [14]. All initial searches were conducted in the Netherlands using the search terms “Heart Rate Variability” and HRV; no additional search filters were applied. Searches were conducted on both smartphone apps and web-based versions of the app store, with the exact same search terms. All identified apps were recorded in a spreadsheet. Duplicates within the same store were removed.

### Selection Criteria

Initial screening was based on the App store information. Irrelevant apps were excluded at this stage. Any potentially relevant apps were retained, and eligibility was determined through website review if required. All apps were independently screened by 2 reviewers according to the inclusion and exclusion criteria outlined below. Any disagreements were solved by the research team through discussion between the 2 reviewers, with a third reviewer available in case of persistent disagreement. The reasons for exclusion were recorded. Apps were eligible for inclusion if they included functionality to record, analyze, or provide feedback on HRV and were available in English. The apps had to measure or display HRV using a smartphone-based PPG via the camera; wearable devices; or an aggregator app that imports, analyzes, or displays HRV from external wearables.

Apps were excluded if they did not feature functionality to record, analyze, or provide feedback on HRV or if they were no longer accessible during final data extraction (February 2026). Additionally, apps were excluded if they focused on nonhuman participants or were not available in English.

### Data Extraction

Data were extracted from the information provided on the app store listing page, screenshots provided, and the app website where relevant. The most recent update of data extraction was done in February 2026. When details were not available or unclear, developers were contacted via email with a request to provide information. If there was no response, a second email was sent within a month. If there was still no response, the data remained unavailable. Apps were downloaded only when app developers instructed us to do so. None of the apps were used for data collection on volunteers. Data extraction was started in the Netherlands but later transitioned to Ireland. The US app stores were used in both locations to maintain consistency across the extracted data. Duplicates between stores were taken together.

We extracted app metadata (name, developer, release year, year of last update, number of downloads in February 2026, cost or payment model, platform [iOS and/or Android], primary measurement tool or aggregator app, and data storage method) and HRV measurement protocol (recording position [eg, supine, seated, and undefined], time of day, standardization procedures [eg, controlled breathing and resting conditions], and HRV measurement recording duration). With regard to data analysis and presentation, information was extracted on metrics derived (time domain; eg, root mean square of successive differences [RMSSD] and SD of normal-to-normal intervals [SDNN]; or frequency domain, such as low frequency (LF) to high frequency (HF) ratio and percentage of successive NN intervals that differ by more than 50 milliseconds) and data presentation (eg, personal trends, detailed parameters, and normal reference ranges). Additional application functionalities included the option to add self-reported stressors (eg, stressful events, caffeine intake, comments, and workout tracker) and reminders, where available.

For all apps, data were extracted on scientific evidence cited, including whether the evidence comprised peer-reviewed literature on HRV physiology or product-specific validation studies. Furthermore, information related to the feedback provided, such as training advice, stress scores, recovery guidance, or recommendations on how to improve HRV, was recorded. A full list of extraction parameters and their descriptions is provided in Multimedia Appendix 1.

### Content Analysis

Only apps with full information available on app metadata and HRV measurement and analysis, representing a transparent subset, were included in the content analysis. Apps available on both stores were reported together in the content analysis, with the first release year and the most recent year of update used for reporting purposes. The data storage statement from the Apple iTunes store was used.

### Quality of Information Provided, Privacy, and Security

To evaluate the information provided to users, credibility criteria were developed from published information on the appraisal of traditional medical literature [15], authorship, attribution, disclosure, and currency. The disclosure criteria were left out; however, a criterion on privacy and security was added. Authorship examined ownership, creators, contributors, and developers, considering professional affiliations, academic credentials, and other relevant expertise. Attribution assessed whether the app or website cited scientific references to support its content. Currency of information verified the date of the most recent update for the app.

Privacy and security information were extracted based on the type of personal data collected and how the data were processed or stored. Information was extracted from both app stores, each of which uses its classification system. From the Google Play Store, apps were categorized based on whether data were only processed locally without being stored or whether they were encrypted in transit. From the iTunes App Store, data handling was categorized as one of the following: no data were stored, data were processed only locally, data were collected but were not linked to the user’s identity, data were collected and linked to identifiable users, or data were used to track users across other platforms (apps and websites).

## Results

### App Selection

**Figure 1.**
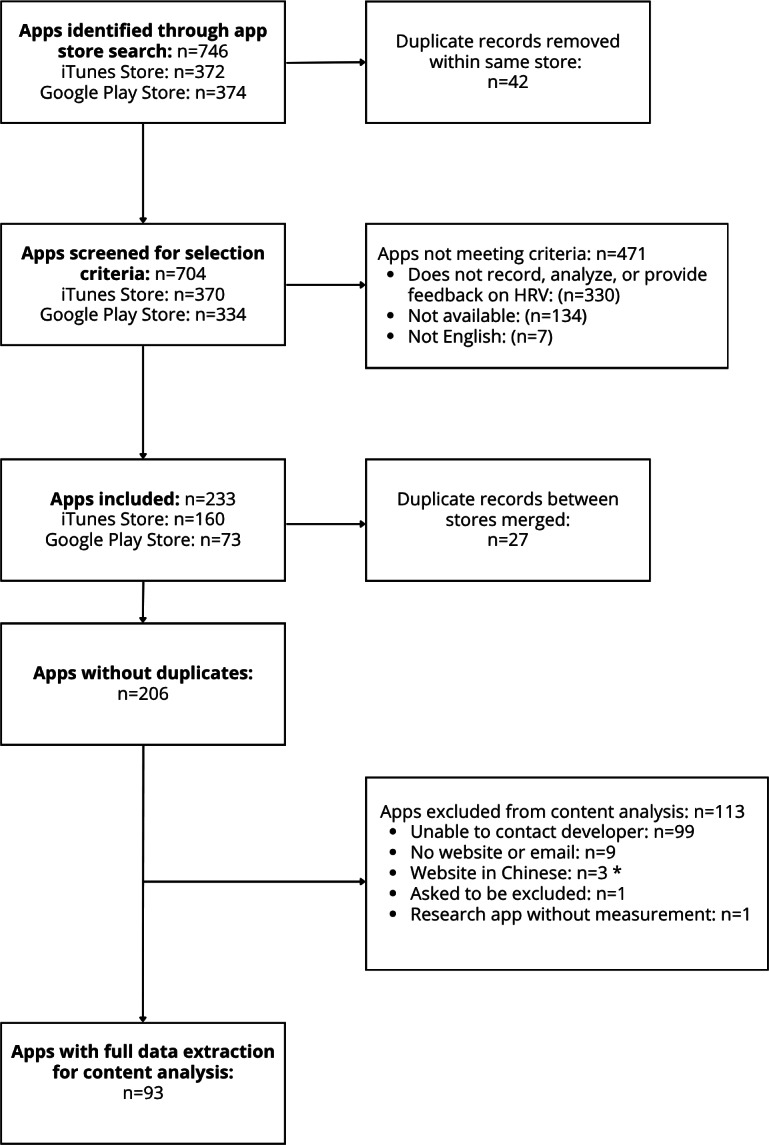
Workflow of the study process. *These apps had an English-language storefront but a Chinese-language developer website, which prevented complete data extraction. HRV: heart rate variability.

The initial search resulted in a total of 746 smartphone apps, of which 42 were within-store duplicates and 27 between-store duplicates. Following eligibility screening, 206 apps were included for initial data extraction (Figure 1). Of these, full information on app metadata and HRV measurement and analysis was obtained from a transparent subset of only 93 unique apps from 80 developers. The app names are listed in Textbox 1. The full data extraction table (N=206) is provided in Multimedia Appendix 2.

Textbox 1. **Transparent subset of apps included in the content analysis (n=93).**

- Acentas HR Monitor
- Athlytic: AI Fitness Coach
- Bevel: All-in-One Health App
- Blood Pressure app BreathNow
- BodyWave: Health tracker
- BradBeat HRV
- Breath Ball: Breathing & HRV
- BreathTuner HRV
- Camera Heart Rate Variability
- CardioBot: Heart Rate Monitor
- Complete HRV
- DailyBeat HRV
- ECG Analysis for Polar H10
- Eclipse Yourself: Health Watch
- Elite HRV: Wellness & Fitness
- ENGY – Health Monitoring based
- Firstbeat Life
- Fitbit
- FITIV Pulse Heart Rate Monitor
- FITTR HART: Smart Ring
- Flowtime: Meditation & Relax
- Garmin Connect
- Gentler Streak Workout Tracker
- Health Hive: Compare Metrics
- Healthye: Heart Rate Monitor
- Heart Analyzer: Pulse Tracker
- Heart Monitor Diary
- Heart Rate Monitor & HRV [BLE]
- Heart Rate Monitor: Cardiobyte
- Heart Rate Monitor: Pulse
- Heart Rate Variability Logger
- HeartBit: Heart Health Tracker
- HeartBreath HRV
- HeartMath
- Heartspace: HRV Training
- HeartWatch: Heart Rate Monitor
- Hera Leto
- HRV Analysis
- HRV Health
- HRV Health basic
- HRV Health Pro
- HRV Trace
- HRV Tracker for Watch
- HRV Train
- HRV Watch: Readiness Score
- HRV4Biofeedback
- HRV4Training
- iHeart HRV
- Inner Balance
- iStress: AI Stress Monitor
- Kana DL
- Kubios HRV
- Lief App
- Livity: Sleep & Health Tracker
- Me - Make health intuitive
- MindBreath – Breathe meditation
- Mindfield eSense
- Moodji: Health & Mood Tracker
- Morpheus Training
- My Autonom Health
- Neuropeak Pro
- ONVY - AI Health Coach
- Optimal HRV
- OtterLife: AI Health Tracker
- Oura
- Paced Breathing
- Polar Flow
- Professional HRV
- Pulse HRV by Camera BLE ECG
- SelfLoops HRV
- Sleep as Android Unlock
- Sleep as Android: Smart alarm
- Sleep Details
- Sonar – Health & Performance
- Stress Check: Health Monitor
- Stress Monitor – Moodpress
- Stress Monitor for Watch
- Stress Tracker: HRV Monitor
- StressEraser Pro
- StressFace: HRV Stress Tracker
- StressWatch: AI Stress Monitor
- Sweetbeat HRV
- SweetBeat HRV Classic
- Training Today
- Ultrahuman
- Visible: Pacing for illness
- Vital Science by Biostrap
- Vitalmonitor
- Wellhero: Stress & Recovery
- Welltory: Health, Heart Rate
- wHealth Dashboard
- WHOOP
- Yudemon HRV

### Metadata on All Apps and Missing Data

Among the 206, a total of 64.6% (n=133) originated from the Apple iTunes Store, 22.3% (n=46) were from the Google Play Store, and 13.1% (n=27) were present in both stores. In total, 63.6% (n=131) of the apps were primary measurement apps, 29.1% (n=60) were aggregators, and 7.3% (n=15) were hybrid, depending on the measurement device used. In total, 70.6% of app metadata and HRV measurement and analysis were found on the website or in the app descriptions, 5.8% were supplied by developers via email, and 23.6% of data were not provided at all.

**Figure 2.**
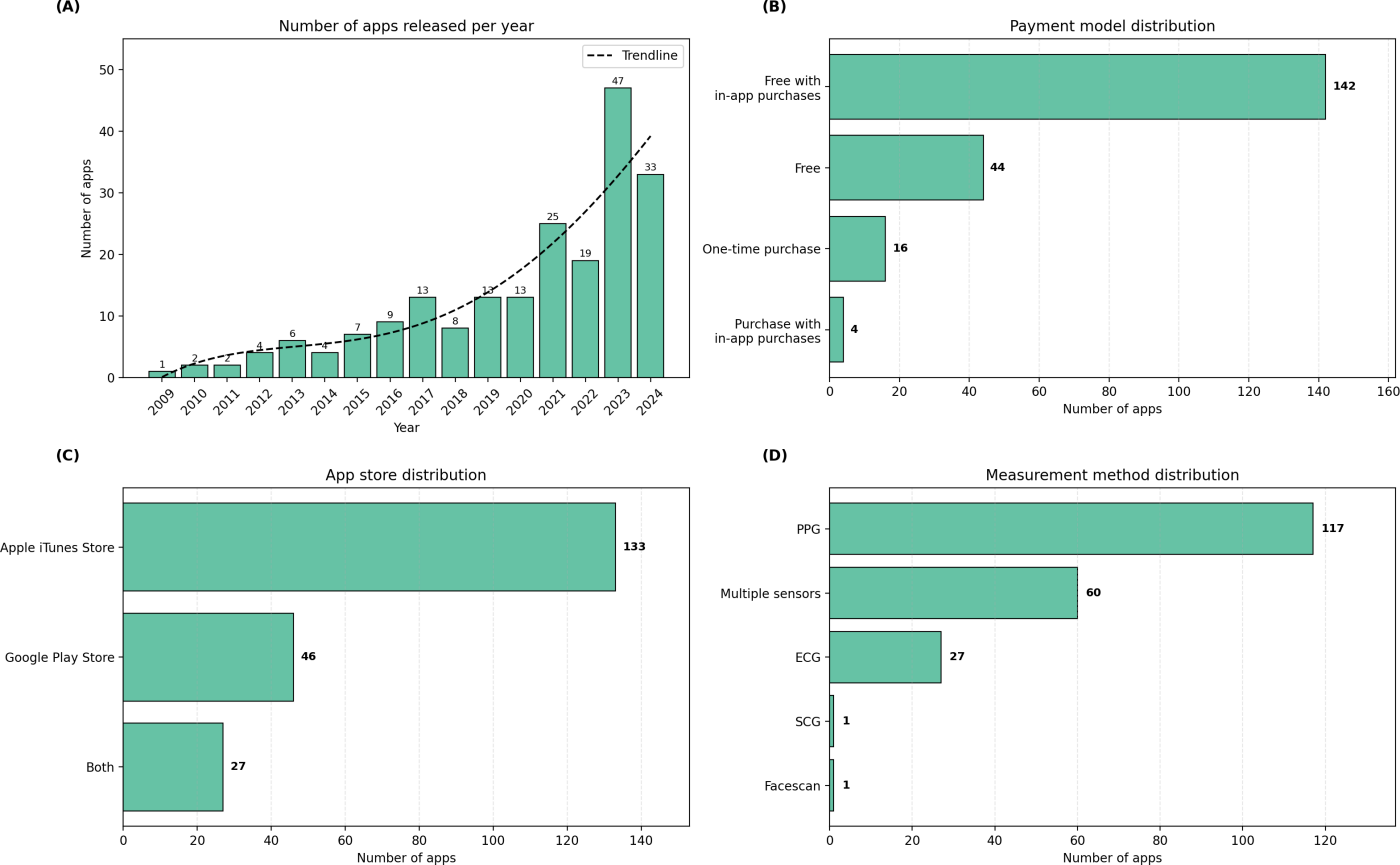
Key characteristics of all smartphone apps (N=206) (A) Bar chart showing the number of apps released per year, with an exponential trendline. Data for 2024 are incomplete because the search was conducted in November 2024. (B) Distribution of the different payment models. (C) Distribution of the app stores. (D) Distribution of measurement methods. ECG: electrocardiography; PPG: photoplethysmography; SCG: seismocardiography.

An overview of the key characteristics of the 206 apps is presented in Figure 2. Of the 206 apps, 205 (99.5%) had information regarding device type, 203 (98.5%) had information regarding sensor type, and 190 (92.2%) described the location of the sensor. However, information about measurement description (n=127, 61.7%) and measurement frequency and duration (n=109, 52.9%) was less frequently available. The majority of apps (n=142, 68.9%) were free with in-app purchases available, and an additional 21.4% (n=44) were completely free (Figure 2). The apps supported various devices. Most apps (n=117, 56.8%) used PPG via a smartphone camera or wearable device for measurements, while 29.1% (n=60) were compatible with multiple sensors that used either PPG and/or ECG. A smaller portion (n=27, 13.1%) relied solely on wearable ECG (chest straps and wireless ECG; Figure 2D).

An exploratory analysis of app download counts indicated that among the most downloaded apps, 40% (n=4) were aggregator apps and 50% (n=5) were primary measurement apps. Most apps relied on PPG sensors (n=7, 70%). As download counts were only available as approximate numbers, these data were analyzed descriptively. Notably, only 3 of these top 10 apps were part of the transparent subset and included in the content analysis, meaning that sufficient data could be extracted from the app stores and associated websites for only these 3 apps. The top 10 apps and their characteristics are presented in Multimedia Appendix 3.

### Content Analysis of Apps With Sufficient Information on HRV Measurement and Analysis

Of the 206 apps, full app metadata and HRV measurement and analysis data were successfully extracted for only 93 apps. These 93 apps formed the basis of the subsequent content analyses. Of the extracted app metadata and HRV measurement and analysis, 88% of the information was obtained from the app descriptions, in the app stores, or linked app websites, while the remaining 12% was collected via email. Complete information could be obtained from publicly available sources for only 49 apps. Stressors (self-reported or workout-tracking data) could be added in 52 apps, while reminder functions were available in 22 apps and unnecessary in 30 apps because measurements were continuous. The summary findings are presented in MultimediaAppendices 4  5.

**Figure 3.**
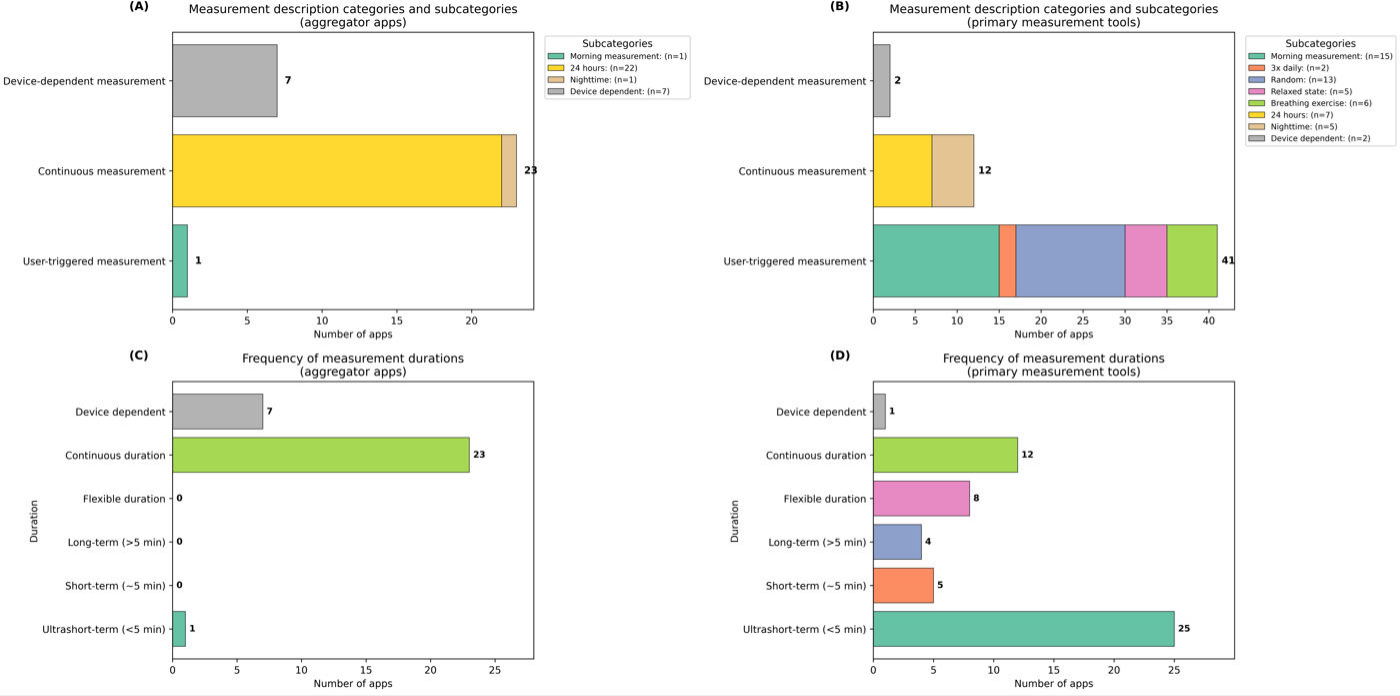
Characteristics from the content analysis stratified by aggregator apps and primary measurement apps: (A) Stacked bar chart showing the frequency of different timing and conditions of measurement for aggregator apps (n=31), (B) stacked bar chart showing the frequency of different timing and conditions of measurement for primary measurement apps (n=55), (C) measurement duration for measuring heart rate variability in aggregator apps (n=31), and (D) measurement duration for measuring heart rate variability in primary measurement apps (n=55). Device dependent indicates that the app collected data from different devices, and the measurement was dependent on the device rather than the app. Hybrid apps are not presented in the figure, as most descriptions and durations depend on the connected device.

In content analysis, PPG was the predominant sensor used for HRV measurement, with 41.9% (n=39) of the apps using PPG signals acquired from smartwatches, smartphone cameras, or PPG devices placed on the finger or ear (Table 1 summarizes sensor locations by sensor type). In total, 58.1% (n=54) of the apps were free with in-app purchases, 24.7% (n=23) were completely free, and the remaining apps required payment, with or without in-app purchases. Most apps used multiple metrics to measure HRV, and the majority used time-domain metrics to report HRV scores such as the RMSSD (n=51), the SDNN (n=48), or the percentage of adjacent normal-to-normal intervals (n=19); other apps also used time-domain metrics and frequency-domain metrics such as HF power (n=22), LF power (n=22), or the LF/HF ratio (n=15). Most measurements were user triggered, with the largest part needing to be completed first thing in the morning (Figures 3A and 3B). Continuous measurements (24 hours or overnight; Figures 3A and 3B) accounted for around 37.6% (n=35). Figures 3C and 3D show the number of apps using ultrashort measurements (<5 minutes), short-term measurements (approximately 5 minutes), and long-term measurements (>5 minutes) [1].

**Table 1.** Cross table showing the sensor used and the sensor location (n=93).

	Arm	Chest	Ear	Finger	Head	Wrist	Multiple sites
Wearable PPG^a^ sensor	1	—^b^	2	7	—	27	2
Bluetooth ECG^c^ HRM^d^	—	19	—	—	—	—	4
Multiple sensors possible	—	2	—	—	2	—	27

a PPG: photoplethysmography.

b Not applicable.

c ECG: electrocardiogram.

d HRM: heart rate monitor.

**Figure 4.**
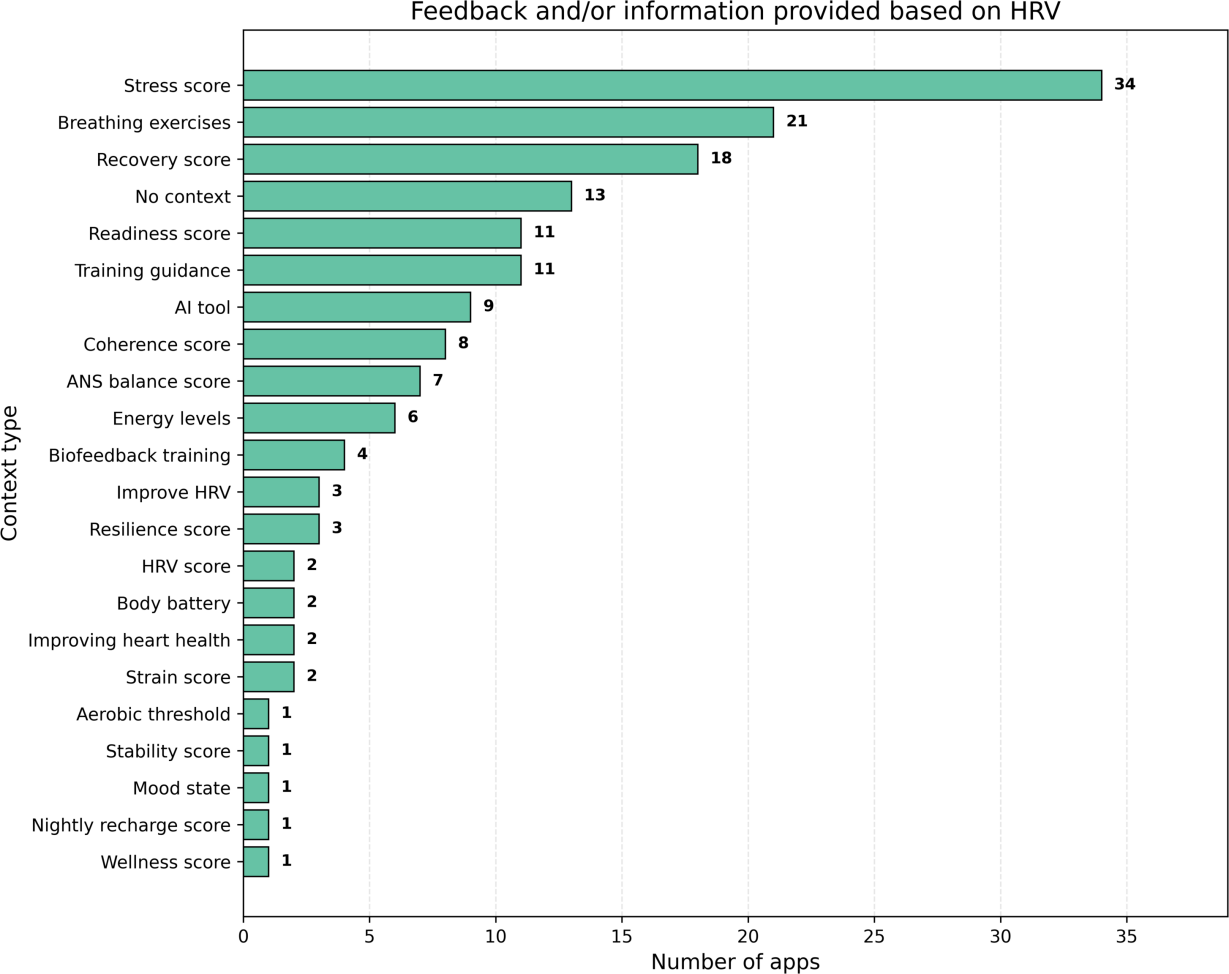
Feedback and information provided by heart rate variability (HRV) apps after measurement. Apps could offer multiple types of feedback (eg, stress score and recovery guidance; n=93). AI: artificial intelligence; ANS: autonomic nervous system.

Most apps (n=76, 81.7%) provide personal trends in HRV scores. Some provide a personalized baseline range or average, calculated as a range over a period of 7 days to multiple months, or present historical data over time. More than half of the apps allowed users to log self-reported stressors (n=52, 66.7%). Scientific references were cited in most cases; the majority cited theoretical references (n=46, 58.2%), while others cited product-specific validation studies supporting the accuracy of the app’s HRV measurements (n=11, 13.9%). Most (n=80, 86%) apps provided contextual HRV insights, guidance, or additional metrics such as recovery or readiness scores (Figure 4). Most apps supported data export, although some provided this functionality only upon request or suggested exporting data through Apple Health. Data storage practices varied: some apps stored no data, while others encrypted it, stored it anonymously, linked it to user identity, or used a combination of these approaches for different types of data.

Apps were stratified into primary measurement apps (n=55, 59.1%), which process raw signals, and aggregator apps (n=31, 33.3%), which use precalculated metrics from third-party hardware or data sources. Hybrid apps (n=7, 7.5%) were those whose functionality depended on the connected device used, allowing them to operate either as primary measurement or aggregator apps. Measurement characteristics also differed between categories. Continuous monitoring was most common among aggregator apps (23/31, 74.2%), while primary measurement apps more frequently used ultrashort, short, or flexible measurement durations (Multimedia Appendix 5).

### Quality Assessment

A clear statement of authorship was provided by 40.9% (38/93) of apps, while 61.3% (n=57) cited peer-reviewed scientific evidence to support their use or information. In terms of information currency, the majority (n=71, 76.3%) were last updated between 2025 and 2026.

Privacy and security of personal data were categorized based on app store classifications. A total of 28 (30.1%) apps collected no data and only processed data locally, 14 (15.1%) apps collected data but did not link it to user identity, 15 (16.1%) apps collected at least some data linked to user identity, 23 (24.7%) apps indicated that personal data may be collected but are encrypted in transit, and 13 (14%) apps indicated that at least some data may be used to track users across other devices. The full results of this quality assessment are available in Multimedia Appendix 6.

## Discussion

### Principal Findings

This study evaluated 206 mobile apps for HRV to categorize their measurement methodologies, feedback mechanisms (eg, stress and recovery scores), and scientific evidence supporting their claims. It is critical to clarify that the content analysis in this manuscript is based on apps with sufficient available data and therefore represents only 45.1% (93/206) of all identified apps, corresponding to the most “transparent” subset of the market. Conversely, most of the market (n=113, 54.9%) remains untransparent and lacks verifiable data regarding its technical protocols and scientific foundations. While the market has seen a steady increase in HRV-related app releases, this study identified a significant lack of transparency regarding how HRV was measured and the scientific evidence supporting the feedback they provided or the claims they made about their utility. Full app metadata and HRV measurement and analysis data could only be successfully extracted from 45.1% (n=93) of the eligible apps, and only 49 apps provided this information on publicly accessible channels.

Among the 93 apps included in the content analysis, the RMSSD was the primary HRV metric in both the scientific literature [16] and in these apps (n=51, 54.8% apps), followed by the SDNN (n=48, 51.6% apps). The prevalence of SDNN might be related to its role as the default metric for Apple Watch–integrated apps [17]. However, this study highlights a discrepancy in measurement validity: while Apple Watch measurements are validated when manually triggered through specific apps (eg, Breath app or Sleep Watch app), values obtained automatically during random daily activities remain unvalidated in current literature [18-20]. Notably, the prevalence of ultrashort recordings (<5 minutes) in the analyzed apps (n=30) prioritizes users’ adherence. While this is scientifically supported for RMSSD, it may compromise the reliability of metrics such as SDNN or frequency-domain powers, which typically require longer durations [1,21]. Furthermore, 86% (80/93) of apps provided contextual insights such as “readiness” or “recovery” scores. These are frequently based on proprietary algorithms that integrate multiple data points, though the underlying scientific evidence for these composite scores was often not explicitly disclosed.

### Comparison With Prior Work

The dominance of PPG in 56.8% (117/206) of the apps aligns with the current trend in wearable technology. While previous research supports the validity of PPG-derived HRV under controlled, resting conditions [7,22,23], the findings of this review suggest that real-world application remains inconsistent. Some smartphone apps that use PPG via phone cameras have undergone scientific validation for HRV measurements [24,25]. Several studies report a good agreement between PPG-derived pulse rate variability and ECG-derived HRV during rest or sleep, particularly in healthy young adults. However, results are less consistent in active, real-world conditions [7], where motion and other uncontrolled conditions can reduce measurement accuracy. Only 31.2% (29/93) of the transparent subset implemented standardization protocols, such as requiring measurement during sleep without distractions (n=6, 6.5%), first thing in the morning after waking (n=17, 18.3%), or during specific resting conditions (n=6, 6.5%).

The accuracy of HRV is influenced by body position and external factors during recording [26,27]; however, only a small proportion of the apps provide specific guidance regarding posture during recording. This lack of standardization may lead to noninterchangeable data, particularly as wearables often tend to underestimate HRV values compared to the gold standard ECG [18,20].

While 61.3% (57/93) of the reviewed apps cited scientific literature to support their general methodology, many proprietary scores, such as body battery, coherence score, readiness, or stress scores, frequently lack the same level of peer-reviewed validation as the raw time-domain metrics (eg, RMSSD) [28]. This review confirms that while the hardware for PPG is increasingly accurate, the software layer providing user feedback often lacks the transparency found in clinical or research settings. Despite identifying 206 HRV apps, only 70.6% of all app metadata and HRV measurement and analysis data were found on the website or in the app descriptions, highlighting a widespread lack of transparency. Additionally, only 2 apps cited authorship, provided scientific attribution, were updated in the last 2 years (currency), and did not collect any data (privacy and security). These factors are crucial for evaluating app credibility, both in the measurement methodologies they use and in the scientific evidence supporting their claims.

### Limitations

First, the terms “HRV” and “Heart Rate Variability” may have excluded wellness apps that measure HRV but do not use this terminology. Second, the search was concluded in November 2024, and although download metrics were updated in February 2026, the sample does not account for new apps released in 2025 or early 2026. Third, content analysis was restricted to 93 (45.1%) apps with full information available on app metadata and HRV measurement and analysis. Results thus represent the market’s transparent subset rather than the broader market. Fourth, 1-time purchase apps were overrepresented in the content analysis compared to the full sample (13/93, 14% vs 16/206, 7.8%), while free apps with in-app purchases were underrepresented (54/93, 58.1% vs 142/206, 68.9%). This skew likely reflects the better resourcing of apps, which allowed for full details to be available and included in the analysis. Fifth, the inclusion of only English-language apps may introduce regional bias and fail to represent the global diversity of the HRV market. Finally, the assessment of privacy and security relied on self-reporting within the app stores, and technical verification (such as network traffic analysis) was not performed. Prior research has identified discrepancies between self-reported and actual data handling practices [29,30]; therefore, caution is warranted when interpreting these findings, as they reflect stated policies rather than verified compliance.

### Future Directions

The findings suggest several avenues for future research and industry development. There is a critical need for the development of standardized reporting frameworks for HRV apps to ensure consistency in how metrics are calculated and displayed to users. Future studies should also focus on evaluating the clinical relevance of the feedback provided by these apps, specifically validating proprietary algorithms against established physiological markers. Additionally, improving the integration of HRV data into cardiovascular research and digital health practices will require developers to enhance data accessibility and transparency regarding their measurement protocols.

### Conclusions

There are currently a large number of HRV apps available, with heterogeneity in the measurement protocols, analysis, and interpretation of HRV. While RMSSD and SDNN are the primary metrics derived, a large portion of apps lacked sufficient information to be included in the content analysis, highlighting limitations in transparency regarding the methods used. Among the included apps, feedback was often provided through proprietary scores, with limited transparency regarding the underlying algorithms or scientific evidence underpinning them.

## Supplementary material

10.2196/84764 Multimedia Appendix 1 Feature description for data extraction.

10.2196/84764 Multimedia Appendix 2 Full dataset used in this study (N=206).

10.2196/84764 Multimedia Appendix 3 Top 10 most downloaded apps in February 2026.

10.2196/84764 Multimedia Appendix 4 Results of the content analysis of apps measuring heart rate variability directly or with wearable devices (n=93).

10.2196/84764 Multimedia Appendix 5 Results of the content analysis split into aggregator apps and primary measurement apps.

10.2196/84764 Multimedia Appendix 6 Results from the quality assessment.

## Abbreviations

ECG: electrocardiogram
HF: high frequency
HRV: heart rate variability
LF: low frequency
PPG: photoplethysmography
PRISMA: Preferred Reporting Items for Systematic Reviews and Meta-Analyses
RMSSD: root mean square of successive differences
SDNN: SD of normal-to-normal intervals
