# Supplementary material for: Mobile Apps for Heart Rate Variability: App Store Search and Content Analysis
Source: JMIR Cardio. 2026 Jul 17;10:e84764. doi: 10.2196/84764 (PMC13378409; doi:10.2196/84764)
Supplement: Multimedia Appendix 1 [file cardio-v10-e84764-s001.docx]

**Multimedia Appendix 1 -** Feature description for data extraction

**Application meta-data:**

1. **Application name:** Name of the app as it appears on the store.
2. **Developer:** Developer names as they appear on the store.
3. **Year of release:** Release year (on the store or on appfigures.com)
4. **Year of last update:** Most recent update (extracted February 2026; appfigures.com)
5. **Number of downloads:** Total downloads as of February 2026, (appfigures.com)
6. **Platform/store:** iTunes app store, Google Play store, or both.
7. **Payment model:** Payment model for the app as it appears on the store.

- Free
- Free with in-app purchases
- One-time Purchase
- Purchase with in-app purchases

1. **App type**:
   1. Primary measurement
   2. Aggregator
   3. Hybrid
2. **Data storage method:** What data storage method is given by the developers in the app store.

- Data not collected
- Data collected but is encrypted in transit.
- Data collected and is not encrypted in transit.
- Data collected but not linked to your identity.
- Data collected and linked to your identity.
- Collection of both identifiable and non-identifiable data.
- Cross-application tracking permitted; identifiable data may be collected.
- Cross-application tracking permitted; non-identifiable data may be collected.
- Cross-application tracking permitted; identifiable and non-identifiable data may be collected.
- No details provided

**HRV Measurement and analysis:**

1. **Device type:** Specific device used for measuring HRV as described in the Appstore or website. This includes
   1. Watch
   2. Ring
   3. Chest strap
   4. Phone camera
   5. Other (specify)
2. **Sensor Type:** What is the type of sensor?
   - PPG: Bluetooth Photoplethysmography sensor
   - ECG: Electrocardiogram sensor
   - SCG: seismocardiography sensor
   - Multiple sensors/device dependent.
3. **HRV Sensor location:** Where on the body the device is placed to record HRV (e.g. finger, wrist, chest, ear, or multiple/device dependent).
4. **HRV Measurement protocol:**
   1. Recording position (e.g. supine, seated, undefined)
   2. Time of day
   3. Standardization procedures (e.g., controlled breathing, resting conditions)
   4. HRV measurement / Recording duration: The description on how long the measurement should be conducted
5. **Recording duration:**
   - - Continuous: measurement is continuously
     - Device dependent: duration is dependent on device used
     - Flexible: duration is flexible in time
     - Long: longer than 5 minutes
     - Short: around 5 minutes
     - Ultra-short: shorter than 5 minutes
6. **Category data displayed:** Category of displayed data:
   - Beat pattern: There is a heartbeat pattern displayed during the measurement
   - Reference range: the results are compared to a normal (non-personal) range
   - Parameters: The results are detailed HRV parameters
   - Personal trends: There are individual trends provided
7. **Metric domain**: Time domain, frequency-domain, non-linear measurements or combination.
8. **HRV metrics derived:** What specific HRV metrics are reported by the application.

**Additional application features:** Additional functionality the app may have

- Self-reported data entry options (e.g., stress, caffeine intake, exercise, notes)
- Reminder functions (availability of measurement reminders if relevant)
- Scientific evidence: Citation of peer-reviewed scientific validation or theoretical scientific citations
- Feedback features (where relevant), including:
  - Stress scores
  - Recovery scores
  - Training recommendations
  - Lifestyle advice
  - Description of algorithms used to generate feedback (if available)
- Export functionality: Is it possible to export the HRV data from the app?
