# Supplementary material for: Mobile Apps for Heart Rate Variability: App Store Search and Content Analysis
Source: JMIR Cardio. 2026 Jul 17;10:e84764. doi: 10.2196/84764 (PMC13378409; doi:10.2196/84764)
Supplement: Multimedia Appendix 3 [file cardio-v10-e84764-s003.docx]

**Multimedia Appendix 2 -** Top 10 most downloaded apps in February 2026

| **Application Name** | **Number of Downloads** | **Aggregator** | **Content analysis** | **Sensor type** |
| --- | --- | --- | --- | --- |
| Da Fit | 960K | No | No | PPG |
| Garmin Connect | 850K | No | Yes | PPG |
| StressWatch: AI Stress Monitor | 450K | Yes | Yes | PPG |
| BetterMe: Health Coaching | 440K | No | No | PPG |
| Heart Rate Monitor – Pulse App | 400K | No | No | Phone PPG |
| iCardiac: Heart Rate & Health | 330K | Depends on device | No | Multiple sensors |
| Oura | 290K | No | Yes | PPG |
| Habit Tracker | 270K | Yes | No | Multiple sensors |
| Health Connect | 270K | Yes | No | Multiple sensors |
| Samsung Health | 260K | Yes | No | PPG |
