# Supplementary material for: Mobile Apps for Heart Rate Variability: App Store Search and Content Analysis
Source: JMIR Cardio. 2026 Jul 17;10:e84764. doi: 10.2196/84764 (PMC13378409; doi:10.2196/84764)
Supplement: Multimedia Appendix 4 [file cardio-v10-e84764-s004.docx]

**Multimedia Appendix 3 -** Results of content analysis of apps measuring HRV directly or with wearable devices (n = 93)

| Characteristics | Number (%) |
| --- | --- |
| **Year of app release**  2011  2012  2013  2014  2015  2016  2017  2018  2019  2020  2021  2022  2023  2024 | 2 (2.2%)  2 (2.2%)  6 (6.5%)  4 (4.3%)  4 (4.3%)  7 (7.5%)  5 (5.4%)  4 (4.3%)  5 (5.4%)  8 (8.6%)  12 (12.9%)  8 (8.6%)  15 (16.1%)  11 (11.8%) |
| **App type**  Aggregator  Primary measurement tool  Hybrid | 31 (33.3%)  55 (59.1%)  7 (7.5%) |
| **Payment model**  Free  Free with in-app purchases  One-time Purchase  Purchase with in-app purchases | 23 (24.7%)  54 (58.1%)  13 (14.0%)  3 (3.2%) |
| **How is data displayed**  Beat pattern  Normal range (non-personal)  Personal trends  Detailed parameters | 6 (6.4%)  6 (6.4%)  76 (81.7%)  5 (5.4%) |
| **HRV metrics reported/displayed to users**  Frequency domain metrics  Time domain metrics  Frequency and time domain metrics  Frequency domain and non-linear measurements  Time domain and non-linear measurements  Time and frequency domain, and non-linear measurements  Heart rate trace | 3 (3.2%)  57 (61.3%)  19 (20.4%)  1 (1.1%)  5 (5.4%)  6 (6.5%)  2 (2.2%) |
