# Supplementary material for: Mobile Apps for Heart Rate Variability: App Store Search and Content Analysis
Source: JMIR Cardio. 2026 Jul 17;10:e84764. doi: 10.2196/84764 (PMC13378409; doi:10.2196/84764)
Supplement: Multimedia Appendix 5 [file cardio-v10-e84764-s005.docx]

**Multimedia appendix 4** - Results of content analysis split into aggregator apps and primary measurement apps

| Characteristics | Aggregator  (n = 31) | Primary measurement  (n = 55) | Hybrid  (n = 7) | Total  (n = 93) |
| --- | --- | --- | --- | --- |
| **Sensor type**  Wearable PPG sensor (e.g. watch/fitness trackers)  Bluetooth ECG HRM  Multiple sensors possible | 24 (25.8%)  -  7 (7.5%) | 14 (15.1%)  23 (24.7%)  18 (19.4%) | 1 (1.1%)  -  6 (6.5%) | 39 (41.9%)  23 (24.7%)  31 (33.3%) |
| **Sensor location**  Arm  Chest  Ear  Finger  Head  Wrist  Multi-site | -  -  -  -  -  24 (25.8%)  7 (7.5%) | 1 (1.1%)  21 (22.6%)  2 (2.2%)  7 (7.5%)  2 (2.2%)  3 (3.2%)  19 (20.4%) | -  -  -  -  -  -  7 (7.5%) | 1 (1.1%)  21 (22.6%)  2 (2.2%)  7 (7.5%)  2 (2.2%)  27 (29.0%)  33 (35.5%) |
| **Measurement duration**  Ultra-short measurement  Short measurement  Long measurement  Flexible duration  Continuous measuring  Device dependent | 1 (1.1%)  -  -  -  23 (24.7%)  7 (7.5%) | 25 (26.9%)  5 (5.4%)  4 (4.3%)  8 (8.6%)  12 (12.9%)  1 (1.1%) | 4 (4.3%)  -  -  -  -  3 (3.2%) | 30 (32.2%)  5 (5.4%)  4 (4.3%)  8 (8.6%)  35 (37.6%)  11 (11.8%) |

PPG = photoplethysmography, ECG = electrocardiogram, HRM = heart rate monitor
