# Supplementary material for: Mobile Apps for Heart Rate Variability: App Store Search and Content Analysis
Source: JMIR Cardio. 2026 Jul 17;10:e84764. doi: 10.2196/84764 (PMC13378409; doi:10.2196/84764)
Supplement: Multimedia Appendix 6 [file cardio-v10-e84764-s006.docx]

**Multimedia appendix 5 -** Results from the quality assessment

| **Application name** | **Authorship** | **Attribution** | **Privacy and Security** | **Currency** |
| --- | --- | --- | --- | --- |
| Training Today | Yes | Yes | 1 | 2026 |
| Vital Science by Biostrap | Yes | Yes | 1 | 2025 |
| Complete HRV | Yes | Yes | 1 | 2024 |
| HRV Health basic | Yes | Yes | 1 | 2022 |
| Paced Breathing | Yes | Yes | 2 | 2026 |
| HeartMath | Yes | Yes | 2 | 2025 |
| ENGY - Health Monitoring based | Yes | Yes | 2 | 2024 |
| Kana DL | Yes | Yes | 2 | 2023 |
| My Autonom Health | Yes | Yes | 2 | 2020 |
| Yudemon HRV | Yes | Yes | 3 | 2026 |
| Livity: Sleep & Health Tracker | Yes | Yes | 3 | 2026 |
| iHeart HRV | Yes | Yes | 3 | 2025 |
| Camera Heart Rate Variability | Yes | Yes | 3 | 2025 |
| HRV Health Pro | Yes | Yes | 3 | 2024 |
| Professional HRV | Yes | Yes | 3 | 2023 |
| WHOOP | Yes | Yes | 4 | 2026 |
| Visible: Pacing for illness | Yes | Yes | 4 | 2026 |
| Ultrahuman | Yes | Yes | 4 | 2026 |
| Oura | Yes | Yes | 4 | 2026 |
| HRV4Training | Yes | Yes | 4 | 2026 |
| HRV4Biofeedback | Yes | Yes | 4 | 2026 |
| Firstbeat Life | Yes | Yes | 4 | 2026 |
| BradBeat HRV | Yes | Yes | 4 | 2026 |
| Sweetbeat HRV | Yes | Yes | 4 | 2025 |
| Kubios HRV | Yes | Yes | 4 | 2025 |
| Inner Balance | Yes | Yes | 4 | 2025 |
| DailyBeat HRV | Yes | Yes | 4 | 2025 |
| HRV Health | Yes | Yes | 4 | 2024 |
| SweetBeat HRV Classic | Yes | Yes | 4 | 2023 |
| Welltory: Health, Heart Rate | Yes | Yes | 5 | 2026 |
| ONVY - Health Coaching with AI | Yes | Yes | 5 | 2026 |
| Blood Pressure app BreathNow | Yes | Yes | 5 | 2025 |
| FITTR HART: Smart Ring | Yes | No | 2 | 2026 |
| Morpheus Training | Yes | No | 2 | 2024 |
| CardioBot: Heart Rate Monitor | Yes | No | 3 | 2025 |
| Sonar - Health & Performance | Yes | No | 4 | 2026 |
| Bevel: All-In-One Health App | Yes | No | 4 | 2026 |
| Heart Rate Monitor: Cardiobyte | Yes | No | 5 | 2025 |
| HeartBit: Heart Health Tracker | No | Yes | 1 | 2026 |
| Heart Rate Variability Logger | No | Yes | 1 | 2026 |
| BreathTuner HRV | No | Yes | 1 | 2026 |
| Breath Ball: Breathing & HRV | No | Yes | 1 | 2026 |
| Athlytic: AI Fitness Coach | No | Yes | 1 | 2026 |
| Sleep as Android Unlock | No | Yes | 1 | 2025 |
| Mindfield eSense | No | Yes | 1 | 2025 |
| HRV Analysis | No | Yes | 1 | 2024 |
| StressEraser Pro | No | Yes | 1 | 2023 |
| Sleep as Android: Smart alarm | No | Yes | 2 | 2026 |
| ECG Analysis for Polar H10 | No | Yes | 2 | 2026 |
| Vitalmonitor | No | Yes | 2 | 2025 |
| Heart Rate Monitor & HRV [BLE] | No | Yes | 2 | 2025 |
| Flowtime: Meditation & Relax | No | Yes | 2 | 2025 |
| Moodji: Health & Mood Tracker | No | Yes | 3 | 2026 |
| Heartspace: HRV Training | No | Yes | 3 | 2024 |
| Wellhero: Stress & Recovery | No | Yes | 4 | 2026 |
| Polar Flow | No | Yes | 4 | 2026 |
| Heart Monitor Diary | No | Yes | 4 | 2026 |
| Lief App | No | Yes | 4 | 2025 |
| Elite HRV: Wellness & Fitness | No | Yes | 4 | 2024 |
| Hera Leto | No | Yes | 4 | 2022 |
| StressWatch: AI Stress Monitor | No | Yes | 5 | 2026 |
| Healthye: Heart Rate Monitor | No | Yes | 5 | 2026 |
| Neuropeak Pro | No | Yes | 5 | 2025 |
| Stress Monitor - Moodpress | No | No | 1 | 2026 |
| Sleep Details | No | No | 1 | 2026 |
| Optimal HRV | No | No | 1 | 2026 |
| Me - Make health intuitive | No | No | 1 | 2026 |
| HRV Watch: Readiness Score | No | No | 1 | 2026 |
| HRV Tracker for Watch | No | No | 1 | 2026 |
| HeartBreath HRV | No | No | 1 | 2026 |
| Heart Analyzer: Pulse Tracker | No | No | 1 | 2026 |
| wHealth Dashboard | No | No | 1 | 2025 |
| HeartWatch: Heart Rate Monitor | No | No | 1 | 2025 |
| Eclipse Yourself: Health Watch | No | No | 1 | 2025 |
| Stress Check: Health Monitor | No | No | 1 | 2024 |
| HRV Train | No | No | 1 | 2023 |
| HRV Trace | No | No | 1 | 2023 |
| acentas HR Monitor | No | No | 1 | 2023 |
| Fitbit | No | No | 2 | 2026 |
| Pulse HRV by Camera BLE ECG | No | No | 2 | 2025 |
| MindBreath - Breathe meditation | No | No | 2 | 2024 |
| SelfLoops HRV | No | No | 3 | 2026 |
| Health Hive: Compare Metrics | No | No | 3 | 2026 |
| StressFace: HRV Stress Tracker | No | No | 3 | 2025 |
| Stress Tracker: HRV Monitor | No | No | 3 | 2024 |
| iStress: AI Stress Monitor | No | No | 3 | 2024 |
| Garmin Connect | No | No | 4 | 2026 |
| Stress Monitor for Watch | No | No | 5 | 2026 |
| OtterLife: AI Health Tracker | No | No | 5 | 2026 |
| Gentler Streak Workout Tracker | No | No | 5 | 2026 |
| FITIV Pulse Heart Rate Monitor | No | No | 5 | 2026 |
| BodyWave: Health tracker | No | No | 5 | 2026 |
| Heart Rate Monitor: Pulse | No | No | 5 | 2025 |

*Privacy & Security scoring: 1. Data not collected, 2. Personal info may be collected but encrypted in transit, 3. Data collected but not linked to you, 4. Data linked to you, 5. Data may be used to track you across apps and websites
